# Dyspnea in Post-COVID Syndrome following Mild Acute COVID-19 Infections: Potential Causes and Consequences for a Therapeutic Approach

**DOI:** 10.3390/medicina58030419

**Published:** 2022-03-12

**Authors:** Klaus J. Wirth, Carmen Scheibenbogen

**Affiliations:** 1 Institute of General Pharmacology and Toxicology, University Hospital Frankfurt am Main, Goethe-University, Theodor-Stern Kai 7, 60590 Frankfurt am Main, Germany; wirth-kriftel@t-online.de; 2 KOSA Pharma GmbH, 60590 Frankfurt am Main, Germany; 3 Institute of Medical Immunology, Charité—Universitätsmedizin Berlin, Corporate Member of Freie Universität Berlin, Humboldt-Universität zu Berlin, and Berlin Institute of Health, 13353 Berlin, Germany

**Keywords:** post-COVID syndrome, long COVID, myalgic encephalomyelitis/chronic fatigue syndrome, dyspnea, hyperventilation, hypocapnia, respiratory alkalosis, sodium overload, calcium overload, mitochondrial dysfunction

## Abstract

Dyspnea, shortness of breath, and chest pain are frequent symptoms of post-COVID syndrome (PCS). These symptoms are unrelated to organ damage in most patients after mild acute COVID infection. Hyperventilation has been identified as a cause of exercise-induced dyspnea in PCS. Since there is a broad overlap in symptomatology with myalgic encephalomyelitis/chronic fatigue syndrome (ME/CFS), causes for dyspnea and potential consequences can be deduced by a stringent application of assumptions made for ME/CFS in our recent review papers. One of the first stimuli of respiration in exercise is caused by metabolic feedback via skeletal muscle afferents. Hyperventilation in PCS, which occurs early on during exercise, can arise from a combined disturbance of a poor skeletal muscle energetic situation and autonomic dysfunction (overshooting respiratory response), both found in ME/CFS. The exaggerated respiratory response aggravating dyspnea does not only limit the ability to exercise but further impairs the muscular energetic situation: one of the buffering mechanisms to respiratory alkalosis is a proton shift from intracellular to extracellular space via the sodium–proton-exchanger subtype 1 (NHE1), thereby loading cells with sodium. This adds to two other sodium loading mechanisms already operative, namely glycolytic metabolism (intracellular acidosis) and impaired Na^+^/K^+^ATPase activity. High intracellular sodium has unfavorable effects on mitochondrial calcium and metabolism via sodium–calcium-exchangers (NCX). Mitochondrial calcium overload by high intracellular sodium reversing the transport mode of NCX to import calcium is a key driver for fatigue and chronification. Prevention of hyperventilation has a therapeutic potential by keeping intracellular sodium below the threshold where calcium overload occurs.

## 1. Introduction

Dyspnea, shortness of breath, and chest pain are frequent symptoms of the post-COVID syndrome (PCS) [1,2,3]. Recent studies in PCS suggest that there is a broad overlap in symptomatology with myalgic encephalomyelitis/chronic fatigue syndrome (ME/CFS), which in the majority of patients may be a post-infectious disease, mostly after viral infections [4,5,6,7,8,9,10]. Both syndromes present with a confusing variety of symptoms with mental and muscular fatigue, cognitive impairment, exertion intolerance with post-exertional malaise (PEM), chronic muscle pain, and headaches, as described for ME/CFS [11].

### 1.1. Hyperventilation as the Cause of Dyspnea in Post-COVID Syndrome Patients with Mild Acute COVID-19 Infections

The presence of dyspnea and cough is easily comprehensible in patients with severe acute COVID-19 infections, due to the organ damage in patients, affecting the lung, heart, and vessels. However, it is surprising to see that dyspnea also occurs in patients with only mild infections [1,2,3,12]. Due to the absence of abnormalities in pulmonary function tests or imaging, the causes of dyspnea and cough are enigmatic in contrast to severe COVID, in which persistent dyspnea due to lung damage and restricted function can be well explained. There must be a fundamental difference in the causes of dyspnea in both cohorts, although it cannot be excluded that, in patients with severe infection and organ damage, the enigmatic mechanisms of the cohort with mild acute infections are also (additionally) operative. The latter cohort will be the topic of this paper.

In an attempt to understand the mechanism of dyspnea in PCS patients with mild acute infections, cardiopulmonary exercise testing (CPET) was performed in several studies [1,2,3]. All report the occurrence of hyperventilation during exercise. There were no abnormalities in blood gases or any other pulmonary or cardiovascular parameters that could explain hyperventilation. A remarkable finding was that all patients increased their ventilation and their tidal volume as soon as the effort started [2]. It is an excessive stimulation of the respiratory center in the brain stem that causes the feeling of air hunger (dyspnea).

### 1.2. The Potential Causes of Hyperventilation

Hyperventilation at rest can be psychogenic, but it is unlikely that this is the cause of hyperventilation during exercise in PCS. The early stimulation of respiration in PCS would fit with the known early physiological stimulation of respiration by skeletal muscle afferents [13], both by metabolic and movement sensors. The earliest, blood gas independent respiratory stimuli in exercise, are central commands and inputs from skeletal muscle afferents.

Since many PCS patients display largely overlapping symptomatology with ME/CFS and a subset fulfills diagnostic criteria for ME/CFS [6,7,14] we attempt to explain the causes for dyspnea and other symptoms by a stringent application of assumptions described for ME/CFS in recent review papers [15,16,17].

In ME/CFS, the poor metabolic situations in skeletal muscles are considered due to a combination of malperfusion and a complex mitochondrial disturbance [16]. Both mitochondrial and vascular dysfunctions in skeletal muscles can be explained by dysfunctional ß2-adrenergic receptor (ß2AdR) signaling due to autoantibodies, polymorphisms, or desensitization. Excessive vasoconstriction can be a result of ß2AdR (vasodilator) dysfunction plus endothelial dysfunction of any cause in the presence of a high vasoconstrictor sympathetic tone. In PCS, vascular disturbances are obvious and first studies provide evidence for endothelial dysfunction [18,19]. Moreover, microcirculation could be disturbed by two separate, endothelial-independent mechanisms in PCS: (1) microthrombi may seriously affect microcirculation [20]. (2) Significant changes in lymphocyte stiffness, monocyte, and neutrophil sizes and deformability, and decreased erythrocyte deformability were found and discussed to be connected with long-term symptoms of the recovered patients [21]. These changes may worsen rheological properties and capillary perfusion together with endothelial dysfunction causing tissue and muscle hypoxia. Increased anaerobic glycolytic pathways may cause intracellular acidosis and subsequent activation of the sodium–proton-exchanger subtype 1 (NHE1) to raise intracellular sodium. NHE1 is the most important proton transporter in many tissues, including skeletal muscles and the heart [22,23]. The mechanisms of sodium loading and the role of NHE1 are the focus of this paper and will be explained in detail below. Interestingly, in leukocytes of ME/CFS patients, impedance changes were found in response to an osmotic challenge, which inevitably activate the NHE1, which are not seen in healthy controls [24]. The mechanisms of these changes are not known but could include alterations in membrane phospholipid functions, possibly as a result of altered ATP production, and/or disturbances of volume regulations. In blood cells, NHE1 is the most important volume regulator [25].

The excessive respiratory stimulation arising from the poor metabolic skeletal muscle situation by afferent input can be further enhanced by a central mechanism of autonomic dysfunction. Dysautonomia characterized by abnormal heart rate variability is present in PCS with fatigue [26]. A central command as the first mechanism of respiratory stimulation can be enhanced as part of the autonomic disturbance. This term describes the simultaneous activation of central somatomotor and autonomic descending pathways [27]. It means that the central neural drive to exercise also stimulates ventilation and raises blood pressure and heart rate. Orthostatic stress and a decrease in cerebral blood flow are strong causes for adrenergic hyperactivity as outlined in our previous paper [17]. Orthostatic symptoms and reduced cerebral blood flow are similar in PCS and ME/CFS [14]. Reduced cerebral blood flow makes a strong contribution to mental fatigue, cognitive dysfunction, and dysautonomia [17].

Taken together, it can be assumed that the respiratory stimulating mechanism by skeletal muscle afferents in the working muscle in PCS occurs earlier and is more intense due to a possible poor metabolic situation compared with the healthy state, thereby causing an excessive respiratory drive. Dyspnea in PCS therefore does not occur because of abnormal blood gases, but as a result of the altered metabolic situation in skeletal muscles. Moreover, in PCS, stimulation of the respiratory center may be further enhanced by dysautonomia, resulting in a significant hyperventilation that leads to hypocapnia and respiratory alkalosis [28].

### 1.3. Possible Consequences of Hyperventilation and Respiratory Alkalosis on Muscular Ionic Homeostasis

Hyperventilation aggravates the skeletal muscle metabolic situation via respiratory alkalosis and the buffering mechanisms that it entails, with serious consequences for both the acute metabolic or energetic situations and the chronification of the disease. One of those buffering mechanisms is a shift of protons from the intracellular to the extracellular spaces, causing cellular sodium loading by the activity of the NHE1 [22,23]. We consider sodium overload in skeletal muscles as key in the pathophysiology of ME/CFS. We assume insufficient stimulation of the skeletal muscles Na^+^/K^+^ATPase together with high NHE1 activity as the critical mechanisms in the skeletal muscle pathophysiology leading to increased sodium uptake and finally to sodium and calcium overload, which probably has the strongest impact on mitochondrial function. [15,16]. The skeletal muscle Na^+^/K^+^ATPase is physiologically stimulated by epinephrine (via ß2AdR, which are dysfunctional), and calcitonin-gene related peptide (CGRP). Interestingly, small fiber neuropathy, which is present in a subset of patients with ME/CFS, is found in patients with PCS, too [29]. We believe that small fiber neuropathy contributes to the chronification of the disease by a lack of the vasodilatory neuropeptides substance P and CGRP and insufficient stimulation of Na^+^/K^+^ATPase by the latter. The mechanisms of sodium loading are shown in Table 1.

With the recognition that hyperventilation is the cause of dyspnea in PCS patients with mild acute infections, the potential consequence of respiratory alkalosis for intracellular sodium loading by the NHE1 and for cellular and mitochondrial calcium dynamics has to be considered. The question is whether this mechanism is quantitatively important enough to be of pathophysiological relevance.

Integrating the finding of hyperventilation in PCS patients into the pathophysiological concept of ME/CFS can explain key symptoms and disease chronification. Hyperventilation, as observed already very early during exercise in PCS patients, will cause respiratory alkalosis, which will be immediately mitigated by activation of various buffering mechanisms, including activation of the NHE1 exchanger, which extrudes protons from the intracellular compartment into the extracellular space in exchange for the cellular uptake of sodium. The proton gradient between extracellular and intracellular spaces, which suddenly steepens by hyperventilation, activates the NHE1 without delay (only by the gradient). In PCS or ME/CFS, the proton gradient could become very steep for two reasons: by a high intracellular proton generation as consequence of the energetic disturbance (intracellular acidosis) and by the hyperventilation-induced extracellular alkalosis. This will strongly activate the NHE1, thereby loading the myocytes with sodium already early on during exercise, with negative consequences for intracellular calcium homeostasis, as will be explained below in more detail.

In skeletal muscles and the heart, NHE1 is the most important proton transporter [16,22], and its activation will result in intracellular sodium loading and in calcium overload via the reverse mode of the sodium–calcium-exchanger (NCX) to cause functional damage, as the most severe scenario. At the physiological intracellular sodium concentration, NCX operates in the forward mode, exporting calcium [30]. High intracellular sodium concentration reverses the transport mode to then import calcium.

**Figure 1:**
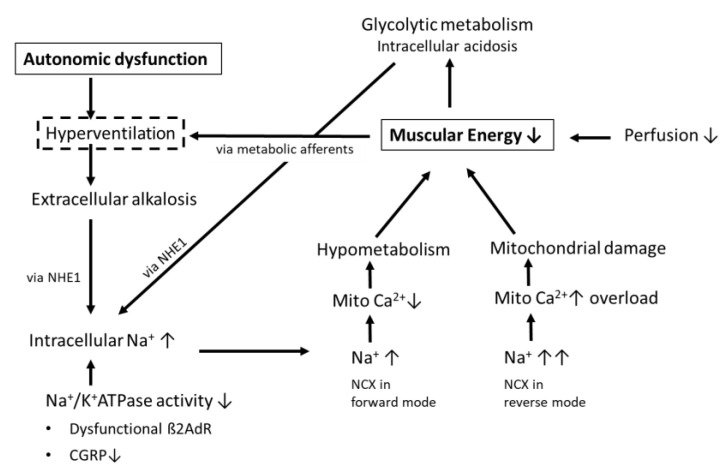
Pathophysiological changes in skeletal muscles related to exercise-induced hyperventilation in PCS.

Mitochondrial metabolism is driven by mitochondrial calcium and mitochondrial dysfunction could arise from either mitochondrial calcium overload, leading to functional damage, or from mitochondrial calcium deficiency, causing under-stimulation of mitochondrial energy production, hypometabolism, and muscular fatigue. We believe that the two states can exist sequentially in individual patients with PCS and ME/CFS, and that the shift between both states is critically dependent on the transport direction of the NCX (Figure 1).

Mitochondrial calcium overload causes functional damage or minimal structural damage, which is not readily reversible. We believe it occurs when intracellular sodium rises to the extent where NCX reverses its transport mode to import calcium to cause calcium overload, the paradigm of the ischemia–reperfusion injury, which also affects the endothelium. This causes more severe and longer lasting PEM as the functional damage takes time to resolve. One of the consequences of mitochondrial calcium overload could be desensitization to calcium in the mitochondrium.

Mitochondrial calcium deficiency causes under-stimulation of mitochondrial metabolism leading to muscle fatigue. It could occur while the NCX is still in the forward mode at moderately elevated sodium levels. This is because elevated intracellular sodium also increases the driving force of the mitochondrial sodium–calcium-exchanger, a transporter different from the sarcolemmal NCX and abbreviated as NCLX, which exports calcium from the mitochondrium creating a calcium unloading effect, which is predominant over the loading effect in the forward mode of the sarcolemmal NCX to lower mitochondrial calcium [16].

If mitochondrial calcium is low for a longer time, this may compensatorily lead to mitochondrial hypersensitivity to calcium to improve the metabolic situation. It would (insidiously) prepare the conditions for the next hit to the mitochondrium by calcium overload when the NCX suddenly turns into the reverse mode at high intracellular sodium during exercise. Mitochondrial hypersensitivity to calcium could explain why moderate calcium overload resulting from small efforts may already cause functional or minimal structural damage to the mitochondrium. Back in the forward mode again, at rest, mitochondrial calcium gets low, as described above, and coincides with functional mitochondrial damage. Together, this causes an energy crisis (crash). A new cycle begins. Thus, cycling of mitochondrial calcium dynamics (calcium concentrations and calcium sensitivity) could explain the varying pattern of symptoms and severity over time (phases of improvement or relative recovery alternating with crashes) and keep the patient caught in the disease.

Hyperventilation and associated alkalosis in PCS could thus contribute to the energetic skeletal muscle disturbance by raising intracellular sodium via the role of NHE1-activity in buffering the alkalosis as one of three mechanisms raising intracellular sodium (Figure 1). We believe that the extent of hyperventilation seen in PCS would be harmless if the Na^+^/K^+^ATPase in skeletal muscles worked properly, if there was no metabolic disturbance activating the NHE1, and no preexisting functional damage (including hypersensitivity to calcium). The disturbance in mitochondrial calcium and the subsequent energetic deficit induced or enhanced by hyperventilation in PCS, which is also seen in ME/CFS [31], could even more stimulate respiration and alkalosis early on in exercise as explained above (vicious circle). Thus, according to our hypothesis, the respiratory disturbance in PCS clinically manifesting as dyspnea does not only reduce the patient’s ability to exercise. It could also be of considerable pathophysiological relevance for the mechanisms involved in the chronification of the disease. We assume that the sodium-induced calcium overload, which not only affects the mitochondrium but also the endothelium through reactive oxygen species (ROS), lowers the threshold for the next event of such kind, to an extent that even activities of everyday life can re-induce it thereby perpetuating the disease [16].

In this paper, we attempt to explain the causes of exercise-induced dyspnea in PCS and its consequences. Whether hyperventilation also occurs in PCS at rest, following emotional or mental over-activity and stress, remains to be shown. Hyperventilation and respiratory alkalosis may contribute to PEM after mental or emotional activities via the same mechanisms that we have outlined here for exercise. Respiratory alkalosis following mental or emotional over-activities may constitute another mechanisms contributing to PEM and add to the ones we have already postulated previously [16].

### 1.4. Differential Mechanisms of Dyspnea following Mild and Severe COVID-19

Finally, the recognition that hyperventilation plays an important pathophysiological role in the development of PCS in patients with mild acute infections also enables understanding what is different in the pathophysiology of post-COVID dyspnea between patients with mild and severe acute infections. Patients with restricted lung functions, as a sequel of an acute severe pulmonary infections, will suffer from inadequately low ventilation during exercise causing dyspnea. Although these patients may still hyperventilate, they will not develop hypocapnia and alkalosis, which we see as one of three mechanisms causing intramuscular sodium loading, as they have impaired gas exchange. Therefore, intracellular sodium may remain below the threshold where the NCX changes its transport mode to import calcium, causing calcium overload and damage. Thus, paradoxically, these patients may be protected from this special ionic disturbance in skeletal muscle, by their restricted lung functions. Patients who become quite hypoxic during the acute infection because of a low respiratory drive are also unlikely to develop hyperventilation and respiratory alkalosis.

## 2. Conclusions and Therapeutic Implication

Persistent shortness of breath, fatigue, and cognitive symptoms in PCS are unrelated to organ damage in most patients after mild COVID. Exercise hyperventilation and dysautonomia may explain many symptoms. Since reversal of the transport mode of the NCX, which causes mitochondrial damage in skeletal muscle by calcium overload occurs when sodium rises to a certain concentration, therapeutic efforts to reduce hyperventilation seem worthwhile, in attempt to keep intracellular sodium below the critical sodium threshold for the reverse mode. In psychogenic hyperventilation, respiratory alkalosis can be effectively prevented by dead space breathing raising arterial pCO_2_, but its application is not practical during exercise. There is some indication that respiratory training may improve the clinical situation in PCS [32]. Autonomic dysfunction was shown to be improved by slow deep breathing and hyperoxia in diabetes [33]. Clinical trials with oxygen therapy in long COVID are ongoing.

## Figures and Tables

**Table 1:** Mechanisms of sodium loading in skeletal muscles.

Hyperventilation-induced respiratory (extracellular) alkalosis via NHE1; Intracellular acidosis (glycolytic metabolism) via NHE1; Insufficient stimulation of Na^+^/K^+^ATPase to transport sodium out of myocytes due to dysfunctional ß2AdR and CGRP-deficit (small fiber neuropathy).

## Data Availability

Not applicable.
